# Ownership, coverage, utilisation and maintenance of long-lasting insecticidal bed nets in three Health Districts in Cameroon: a cross-sectional study

**DOI:** 10.11604/pamj.2024.48.85.36061

**Published:** 2024-07-03

**Authors:** Frederick Nchang Cho, Yayah Emerencia Ngah, Ismaila Esa, Patrick Kofon Jokwi, Peter Canisius Kuku Elad, Solange Fri Munguh, Blessing Menyi Cho, Paulette Ngum Fru, Celestina Neh Fru, Tassang Andrew

**Affiliations:** 1 Cameroon Baptist Convention Health Services, HIV Free/Strengthening Public Health Laboratory Systems, Buea, Cameroon,; 2 Infectious Disease Laboratory, Faculty of Health Sciences, University of Buea, Buea, Cameroon,; 3 Central African Network for Tuberculosis, HIV/AIDS and Malaria (CANTAM), University of Buea, Buea, Cameroon,; 4 Catholic School of Health Sciences, Saint Elizabeth Hospital, Shisong, Cameroon,; 5 District Health Services Bamenda, North West Regional Delegation of Health, Ministry of Health, Bamenda, Cameroon,; 6 Department of Microbiology and Parasitology, University of Buea, Buea, Cameroon,; 7 Department of Public Health and Hygiene, University of Buea, Buea, Cameroon,; 8 District Health Services Tiko, South West Regional Delegation of Health, Tiko, Cameroon,; 9 Department of Sociology and Anthropology, Faculty of Management and Social Sciences, University of Buea, Buea, Cameroon,; 10 Atlantic Medical Foundation, Mutengene, Cameroon,; 11 Department of Obstetrics and Gynaecology, Faculty of Health Sciences, University of Buea, Buea, Cameroon,; 12 Buea Regional Hospital Annex, Buea, Cameroon

**Keywords:** Long-lasting insecticidal nets, ownership, universal coverage, utilization, maintenance

## Abstract

Introduction: the Bamenda, Santa and Tiko Health Districts are in the highest malaria transmission strata of Cameroon. The purpose of this study was to explore the indicators of ownership and utilisation as well as maintenance of Long-Lasting Insecticidal Nets (LLINs) in three health districts in Cameroon.

Methods: a cross-sectional household survey involving 1,251 households was conducted in the Tiko Health District (THD) in June and July 2017 and in Bamenda and Santa Health Districts in March to May 2018. A structured questionnaire was used to collect data on LLIN ownership, utilisation, and maintenance as well as demographic characteristics.

Results: the average number of LLINs per household was higher in the Bamenda Health District (BHD) compared to the THD (2.5, range; 0-6 vs. 2.4, range; 0-6) as well as the household ownership of at least one LLIN (93.3% vs. 89.0%). The proportion of the defacto population with universal utilisation was higher in BHD compared to THD (13.1% vs 0.2%). In the multinomial regression analysis, households in the SHD (p = 5.5x10-4, OR; 0.3, 95% C.I; 0.1-0.6), were less likely to own at least one LLIN compared to those in THD. Eighty-seven point one percent (87.1%) of household heads admitted that LLINs could be washed, while 50.1% affirmed the correct washing frequency.

Conclusion: ownership of LLINs was low in the THD in comparison to the goal of one for every two household members. Overall, LLINs coverage and accessibility was still low after the free mass distribution campaigns (MDCs), as only 14.6% of children 0-5 years and 16.1% of the entire population used LLIN the night before the survey.

## Introduction

Malaria is a preventable and curable disease transmitted by the bites of female *Anopheles* mosquitoes [1,2]. It is a serious global public health problem with an estimated 216 million cases in 91 countries in 2016 [2,3]. Africa is the most affected region, with 90% of all estimated malaria cases and 91% of deaths in 2016 and 15 African countries alone contributing 80% of all cases, Nigeria and the Democratic Republic of the Congo (DRC), being the top two contributors [1,3]. Cameroon, bordered by the Gulf of Guinea and Nigeria to the west; Chad and the Central African Republic to the east; and Equatorial Guinea, Gabon, and the DRC to the south, through the Ministry of Health (MOH), completed her second national universal long-lasting insecticidal nets (LLIN) campaign in 2019 [4]. With support from the Global Fund, the MOH has made provision of free LLINs to pregnant women at antenatal care clinics since 2008 [5]. In 2011, the Cameroon MOH undertook a nationwide free LLINs distribution campaign from health facilities to all households, with the objective to provide a LLIN with a lifespan of five years, to all household beds or a LLIN for every two individuals per household, to a maximum of three LLINs per household [6,7]. Despite the distribution of free LLINs, malaria continues to be endemic in Cameroon, with an estimated mortality rate of 11.6%, surpassing that of the African region of 10.4% [5]. It is the first major cause of morbidity and mortality among children under five years and pregnant women [8-11], accounting, respectively, for 18% and 5% of the total population estimated at 19 million [12]. The main contemporary malaria control interventions are insecticide-treated bed nets and indoor residual spraying [1,13,14]. Alliance for Malaria Prevention has been instrumental in keeping LLIN campaigns on track: between 2014 and 2016 about 582 million LLINs were delivered globally and in 2017, there was the successful delivery of over 68 million nets to targeted recipients in sub-Saharan Africa (SSA) and beyond [3]. Over 80% of all households have at least one mosquito net, up from 57% in 2011, still only about 60% of these households have enough nets to cover everyone at night [15]. The proportion of people in SSA sleeping under LLINs rose from less than 2% to over 50% between 2000-2015, preventing an estimated 450 million malaria cases [16]. Most studies in Cameroon and elsewhere have focused on various aspects of net ownership and utilisation. Effective LLIN use in the prevention of malaria in parts of North West Cameroon [17], Plasmodium falciparum infection in Rural and Semi-Urban Communities in the South West Cameroon [6], predictive factors of ownership and utilisation in the Bamenda Health District (BHD) [18], socio-demographic factors influencing the ownership and utilisation among malaria vulnerable groups in the Buea Health District [19] and the qualitative study on the use and maintenance of LLINs in Bouaké - Côte d´Ivoire [20]. However, there is paucity information on the indicators of LLINs ownership/utilisation and maintenance. This study examines the indicators of net ownership/utilisation as well as maintenance, through the analysis of household survey data collected from three health districts in Cameroon.

## Methods

**Study area**: the study area consisted of the BHD with an estimated 350,000 residents and the Santa Health District (SHD), 35 Km from the BHD with 73,406 residents in the North West Region and the Tiko Health District (THD), 351 Km from the BHD with an estimated 134,649 residents in the South West Region of Cameroon [21]. The BHD (a semi-urban community) and the SHD (rural community) are in the high western plateau altitude malaria geographical strata of Cameroon, where malaria transmission is permanent, occurring all year long, sometimes lessened by altitude although never totally absent [12,22]. It is one of the most densely populated regions of Cameroon [10,12]. The THD (urban and rural communities) is in the coastal strata, a zone of dense hydrophile forest and mangrove swamp with the highest transmission of malaria in the country [10,12]. Like the Buea health district, the THD has a constant variation in the trends of malaria prevalence all round the year [23,24].

**Sampling design**: this cross-sectional household survey conducted in the THD from June to July 2017 and BHD and SHD from March to May 2018, utilised a stratified multistage cluster sampling design. The study sampling frame included all health areas (HAs) in the study area, except those that were inaccessible for security reasons. Within each HA, localities were subdivided into quarters (primary sampling units/clusters in our study). On average, each HA had about five quarters.

**First stage**: based on the probability proportionate to size (PPS), we randomly selected four HAs in the THD and conveniently sampled one each from the BHD and SHD.

**Figure 1:**
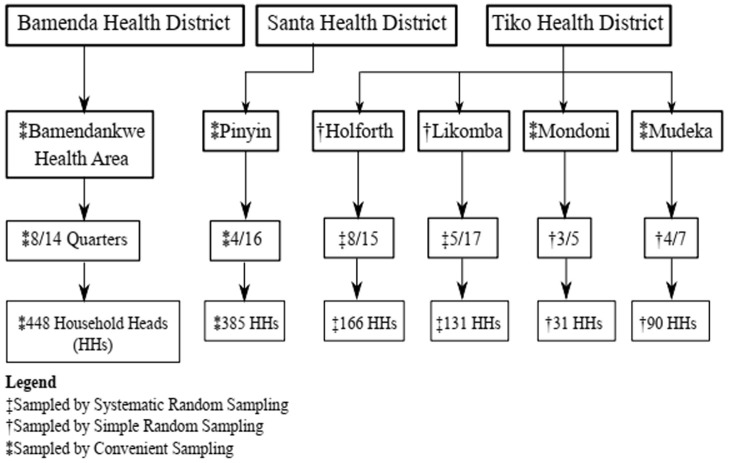
multi-stage sampling

**Second stage**: within each HA, we randomly selected at least three quarters and at most eight quarters by PPS, thus totalling 32 quarters (Figure 1) in the sample.

**Third stage**: within each quarter, households were enumerated and selected by convenient, simple/or systematic random sampling. The estimated number of households in each quarter was obtained from the quarter leader to determine the sampling interval to select the households.

**Study population and target sample size**: the study population consisted of household heads or their representatives who had lived in the household for at least one year; could speak Pidgin, English, or French; and who were ≥ 18 years old. A minimum sample size of 384 for each health district was determined with the Cochrane formula [25]:


$$n=\frac{z^{2}*pq}{d^{2}}$$

Where: n = minimum sample size, Z = 1.96, critical Z value at 95% confidence interval (95% C.I.), p = 50% estimated population of households owning mosquito bed nets = 0.5, q = 1-p = 0.5, (p)(q) = (0.5)2 = 0.25, d = acceptable margin of error for proportion being estimated = 0.05.

**Recruitment procedures and measures:** interviewers explained the purpose of the study and obtained verbal informed consent from the head of the household or spouse. In cases where neither household head was available, any elderly person who has lived in the house for at least the last 12 months replaced him/her.

**Outcome variables:** the main LLIN outcome variables were:

**LLINs ownership indicators:** household LLINs ownership: the proportion of households with at least one LLIN, where the numerator comprises the number of households surveyed with at least one LLIN and the denominator, the total number of households surveyed. Coverage: proportion of households with at least a LLIN for every two persons, where the numerator comprises all households where the ratio between the number of LLINs owned and the number of de-jure members of that household, that is, usual members excluding visitors, is 0.5 or higher and the denominator is the total number of sampled households. Access to LLINs within the household: the proportion of the population with access to LLINs (population that could sleep under a LLIN if each LLIN in the household were used by up to two people) and proportion of the defacto household population that slept under a LLIN last night. Defacto household members are all people present in the household on night of the survey including visitors [8,26].

**LLIN utilisation indicators:** household universal utilisation: proportion of the population that slept under a LLIN the previous night [8,26]. By the vulnerable population in the household: proportion of children under five (or pregnant women) that slept under a LLIN the previous night [8]. Regularly sleeping under bed-nets: household heads who reported habitually using nets daily [27]. Household head slept under: a LLIN last night: the proportion of households in which the household head slept under a LLIN last night, where the numerator comprises the number of households surveyed wherein the household head slept under a LLIN last night and the denominator, the total number of households surveyed.

**Maintenance indicators:** washing LLINs: the proportion of household heads who accepted that LLINs can be washed, where the numerator comprises the number of household heads surveyed who accepted that LLINs can be washed and the denominator, the total number of household heads surveyed. Recommended LLINs washing frequency: proportion of household heads who stated the recommended LLINs washing frequency of ≥2 times per year, where the numerator comprises the number of household heads surveyed who stated the correct frequency and the denominator, the total number of household heads surveyed [28].

**Independent variables (IV):** considered for association with LLIN ownership, use, and maintenance were age, gender, marital status, education, occupation, health district, house type, and household composition.

**Statistical analysis:** we entered the data into, and analysed with IBM-SPSS Statistics 26.0 for windows. The Chi square (χ^2^) test was used to compare socio-demographic characteristics with the health districts and multivariate logistic regression to identify significant correlates of the main outcomes. To control for confounders and avert bias, only the most significant independent covariates were included in the multivariate logistic regression analysis. The significant level was set at < 0.05.

**Ethics statement:** the study obtained approval from the Institutional Review Board of the Faculty of Health Sciences, University of Buea (No: 624-05). Administrative authorisation was obtained from the South West Regional Delegation of Public Health. Informed consent was obtained from all participants and confidentiality was maintained at all steps of data collection.

## Results

**Characteristics of the study participants:** a total of 1,251 household heads was sampled with 5,870 defacto residents across six health areas in three health districts. Of the total household residents counted, 1,267 (21.6%) were children 0-5 years old and 93 (1.6%) were expectant mothers. There were generally more female (68.0%) household heads than males, with a mean age of participants of 36.1 (range; 20-60). The overall mean household (or family) size was 4.7 (range; 1-18) members: 4.6 (range; 1-10) in BHD and 5.0 (range; 1-18) in THD (Table 1). Majority of the houses, 804 (64.3%) were made of cement, while households with 1-2 bedrooms; the mean number of bedrooms 2.0 (range; 1-7) were about 1,141 (91.2%). About 1,116 (89.2%) of the households were located near mosquito breeding sites: farms/gardens, bushes, or pools of water.

**Table 1:** socio-demographic characteristics: by health district

	Health district					
Characteristic	Bamenda	Santa	Tiko	n(%)	χ^2^ /F	p-value
**Age groups (in years)**	n = 448	n = 385	n = 418			
20	9	6	15	30(2.4)	46.48	⁑1.9x10-7
21±30	147	112	199	458(36.6)		
31±40	151	115	96	362 (28.9)		
41±50	74	86	58	218 (17.4)		
51±60	67	66	50	183(14.6)		
Mean age	36.2±9.9	38.0±11.	34.3±11.2	36.1±10.8	12.37	⁑4.8x10-6
**Gender**						
Females	282	288	281	851(68.0)	13.57	⁑1.1x10-3
Males	166	97	137	400(32.0)		
**Marital status**						
Unmarried	176	232	151	559(44.7)	55.46	⁑9.1x10-13
Married	272	153	267	692(55.3)		
**Education**						
NFE + Primary	145	137	204	486(38.8)	66.27	⁑1.4x10-13
Secondary	157	143	170	470(37.6)		
Tertiary	146	105	44	295(23.6)		
**Occupation**						
Unemployed	0	140	113	253(20.2)	900.41	⁑4.6x10-189
Agricultural	163	0	28	191(15.3)		
Unskilled/Household/Domestic	70	245	48	363 (29.0)		
State/Parastatal	33	0	126	159 (12.7)		
Professional	182	0	103	285 (22.8)		
**House type**						
Caraboat	16	0	68	84 (6.7)	292.50	⁑3.3x10-60
Mixed (Block & Plank)	0	44	63	107 (8.6)		
Mud Block	125	131	0	256 (20.5)		
Cement Block	307	210	287	804 (64.3)		
Number of bedrooms						
1-3	410	353	378	1,141 (91.2)	0.48	7.9x10-1
4-7	38	32	40	110 (8.8)		
Mean number of bedrooms	2.0±1.0	1.9±1.1	1.9±1.2	2.0±1.1	0.7±3	⁑4.8x10-1
**Environmental risk factor(s)**	363	364	389	1,116 (89.2)	48.99	⁑2.3x10-11
**Household Composition**						
**Mean number of children < 5**	1.0±1.0	1.2±1.1	0.9±0.9	1.0±1.0	12.7±7	⁑3.3x10-6
**Mean household size**	4.6±2.2	4.5±1.7	5.0±2.5	4.7±2.1	6.7±3	⁑1.3x10-3
**Net Ownership**						
Mean number of nets in HHs	2.5±1.4	2.2± 1.2	⁑3.3x10-6			
**Children 6-17 surveyed**	728	544	704	1,976 (33.7)	5.67	⁑3.6x10-3
**Persons ≥ 18**	835	680	1,019	2,534(43.2)	65.15	⁑1.2x10-27
**Expectant women surveyed**	46	35	12	93(1.6)	9.84	⁑5.8x10-5
**Population surveyed**	2,057	1,724	2,089	5,870	6.73	⁑1.2x10-3
Bed-net: Person ratio	0.5±0.3	0.5±0.3	0.5±0.4	0.5±0.3	1.2±1	3.0x10-1

⁑: Statistically significant (p-value < 0.05), HH: Household, NFE: No Formal Education

**LLINs ownership and coverage:** one thousand one hundred and fifty-seven (1,157) representing (92.5%) of the 1,251 households sampled had at least one LLINs, while 836 (66.8%) had at least one bed-net for every two persons in the household (Table 2). The overall LLIN-to-person ratio was 0.50, that is, one net for every two persons (Table 1), constituting a coverage of 3,913 (66.7%) of the defacto population. The mean number of LLINs in the households was 2.4 (range; 0-6). LLINs ownership and coverage were associated with education and health districts (Table 3), where households in the THD significantly owned fewer nets, while those in the BHD significantly (p = 4.9x10-2) had more coverage than other districts. Coverage was also associated with the gender of the household head and household size (Table 3), where households headed by females (p = 4.2x10-3) and that of household size of 1-4 members (p = 3.2x10-2) significantly influenced coverage than the others. Secondary educational and unskilled occupational status significantly influenced household ownership of nets (p < 0.05).

**Table 2:** LLINs ownership/utilisation and maintenance indicators in association with health districts

	Households						Defacto population in households			
LLINs indicator	n (%)	BHD	SHD	THD	χ^2^	p-value	n (%)	BHD	SHD	THD
**Ownership**		n = 448	n = 385	n = 418						
At least One	1,157 (92.5)	418	367	372	12.23	⁑2.2x10-3	5,577 (95.0)	2,000	1,680	1,897
Coverage	836 (66.8)	387	214	235	120.46	⁑7.0x10-27	3,913 (66.7)	1,893	937	1,083
**Accessibility**	819 (65.5)	363	181	275	105.99	⁑9.6x10-24	4,058 (69.1)	1,825	937	1,296
**Utilisation**										
Children 0 – 5 years	520 (41.6)	250	103	167	72.62	⁑1.7x10-16	859 (14.6)	427	188	244
Entire household	261 (20.5)	198	4	59	250.90	⁑3.3x10-55	942 (16.0)	767	10	165
Regularly by HH	484 (38.7)	87	203	194	112.62	⁑3.5x10-25	1,296 (22.1)	346	297	653
By HH last night	350 (28.0)	152	94	104	12.29	⁑2.1x10-3	705 (12.0)	356	111	238
**Care and maintenance**										
LLINs can be washed	1,089 (87.1)	379	341	369	3.74	1.5x10-1	5,148 (87.7)	1,735	1544	1,869
Correct LLINs washing frequency	634 (50.7)	188	205	241	22.77	⁑1.1x10-5	3,169 (54.0)	904	955	1,310

⁑: Statistically significant (p-value < 0.05), HH: Household, BHD: Bamenda Health District, SHD: Santa Health District, THD: Tiko Health District

**Table 3:** multinomial regression analysis of socio-demographic characteristics in association with LLINs ownership indicators

DV →	Ownership‡ (n = 1,157)		Coverage‡ (n = 836)		Accessibility‡ (n = 819)	
IV →	p-value	OR (95% C.I.)	p-value	OR (95% C.I.)	p-value	OR (95% C.I.)
**Age groups (in years)**						
20	7.0x10-1	†1.3 (0.3 - 5.1)	6.1x10-1	†1.3 (0.5 - 3.0)	⁑1.9x10-2	†3.2 (1.2 - 8.3)
21- 30	6.6x10-1	0.8 (0.4 - 1.8)	8.3x10-1	1.0 (0.6 - 1.5)	8.9x10-1	1.0 (0.7 - 1.6)
31- 40	6.2x10-1	0.8 (0.4 - 1.8)	5.8x10-1	†1.1 (0.7 - 1.7)	7.9x10-2	†1.5 (1.0 - 2.4)
41- 50	2.0x10-1	†1.7 (0.8 - 3.7)	7.8x10-1	0.9 (0.6 - 1.5)	5.5x10-1	†1.2 (0.7 - 1.9)
51- 60	Ref	1.0	Ref	1.0	Ref	1.0
**Gender**						
Female	4.3x10-1	†1.2 (0.7 - 2.0)	⁑4.2x10-3	0.7 (0.5 - 0.9)	2.4x10-1	0.8 (0.6 - 1.1)
Male	Ref	1.0	Ref	1.0	Ref	1.0
**Marital status**						
Unmarried	1.1x10-1	†1.5 (0.9 - 2.4)	⁑3.3x10-2	†1.4 (1.0 - 1.8)	9.7x10-2	†1.3 (1.0 - 1.8)
Married	Ref	1.0	Ref	1.0	Ref	1.0
**Education**						
NFE + Primary	2.7x10-1	†1.5 (0.7 - 3.1)	2.2x10-1	†1.3 (0.9 - 1.8)	1.7x10-1	0.8 (0.5 - 1.1)
Secondary	⁑1.8x10-2	†2.2 (1.1 - 4.4)	1.1x10-1	†1.3 (0.9 - 1.9)	1.4x10-1	0.7 (0.5 - 1.1)
Tertiary	Ref	1.0	Ref	1.0	Ref	1.0
**Occupation**						
Unemployed	⁑2.6x10-2	†2.5 (1.1 - 5.5)	4.6x10-1	†1.2 (0.7 - 1.9)	4.2x10-1	†1.2 (0.7 - 2.1)
Agricultural	⁑2.3x10-2	†2.5 (1.1 - 5.3)	⁑1.8x10-2	†1.8 (1.1 - 3.1)	3.5x10-1	†1.3 (0.8 - 2.1)
Unskilled/Household/Domestic	⁑1.6x10-2	†2.5 (1.2 - 5.5)	⁑1.3x10-2	†1.8 (1.1 - 2.9)	4.8x10-1	†1.2 (0.7 - 2.0)
State/Parastatal	5.3x10-1	†1.3 (0.6 - 3.1)	8.9x10-1	1.0 (0.6 - 1.6)	7.0x10-1	0.9 (0.5 - 1.5)
Professional	Ref	1.0	Ref	1.0	Ref	1.0
**Health District**						
Bamenda	⁑4.9x10-2	0.5 (0.3 - 1.0)	⁑6.6x10-19	0.1 (0.1 - 0.2)	⁑7.7x10-7	0.3 (0.2 - 0.5)
Santa	⁑5.5x10-4	0.3 (0.1 - 0.6)	5.5x10-2	0.7 (0.4 - 1.0)	⁑1.5x10-3	†2.2 (1.3 - 3.5)
Tiko	Ref	1.0	Ref	1.0	Ref	1.0
**House types**						
Caraboat	3.5x10-1	0.7 (0.3 - 1.6)	6.5x10-1	0.9 (0.5 - 1.5)	2.7x10-1	†1.4 (0.8 - 2.5)
Mixed (Block/Plank)	7.3x10-2	0.4 (0.1 - 1.1)	2.1x10-1	0.8 (0.5 - 1.2)	9.0x10-1	1.0 (0.6 - 1.7)
Mud Block	8.2x10-1	†1.1 (0.6 - 2.1)	⁑1.2x10-3	†1.8 (1.3 - 2.6)	5.8x10-1	0.9 (0.6 - 1.3)
Cement Block	Ref	1.0	Ref	1.0	Ref	1.0
**House size**						
1 - 3 bedrooms	7.8x10-1	†1.1 (0.5 - 2.6)	⁑3.2x10-2	†1.7 (1.0 - 2.7)	8.2x10-1	0.9 (0.6 - 1.6)
4 - 7 bedrooms	Ref	1.0	Ref	1.0	Ref	1.0
**House composition**						
0 Children 0 - 5 SHLN	6.1x10-2	†4.6 (0.9 - 22.9)	2.0x10-1	†1.5 (0.8 - 2.7)	3.1x10-1	†1.5 (0.7 - 3.2)
1 - 2 Children 0 - 5 SHLN	7.5x10-1	†1.3 (0.3 - 6.2)	2.5x10-1	†1.4 (0.8 - 2.3)	4.9x10-1	†1.3 (0.6 - 2.6)
3 - 4 Children 0 - 5 SHLN	Ref	1.0	Ref	1.0	Ref	1.0
Family size 1 – 4	3.2x10-1	†1.7 (0.6 - 5.3)	⁑2.0x10-3	0.5 (0.3 - 0.7)	⁑2.1x10-9	†7.2 (3.8 - 13.8)
Family size 5 – 7	7.8x10-1	0.9 (0.3 - 2.6)	6.0x10-2	0.6 (0.4 - 1.0)	5.3x10-1	0.8 (0.4 - 1.5)
Family size ≥ 8	Ref	1.0	Ref	1.0	Ref	1.0

⁑: Statistically significant (p-value < 0.05), †: more likely possible, HH: Household, DV: Dependent variable, IV: Independent variable, SHLN: Slept Home Last Night, NFE: No Formal Education.‡. Reference category is: Yes

**Household accessibility to LLINs:** overall household accessibility to bed nets was 819 (65.5%), with a significant (p = 9.6x10-24) association to health districts (Table 2). Household accessibility (Table 3) to bed nets was associated with the age of the household head, health districts, and family size, where household residents in houses headed by those in the 20 years age group, those in the BHD and SHD significantly (p < 0.05), and those with 1-4 members in the family had more access to LLINs than the other groups. About 4,058 (69.1%) of the defacto population, from 819 (65.5%) of the 1,251 households sampled, had access to LLINs in the household.

Use of LLINs: of the 1,251 households sampled, 520 (41.6%) and 261 (20.5%) were those in which all children 0-5 years and those in which all who slept home last night used bed nets, respectively representing 859 (14.6%) and 942 (16.0%) of the 5,870 de facto population that slept home last night (Table 2). Bed-net utilisation in households with all children 0-5 years and the entire family (Table 4), was associated with age, marital status, education, occupation, and health district where more households with household heads in the 20-30 years age group, unmarried, those with primary educational status, those in the agricultural sector, and those in the BHD (p < 0.05) significantly used nets than the other groups. Bed-net utilisation by the entire family (Table 4) was associated with the age of the household head, health district, composition of the household, where more households with household heads in the 21-30 years group, those in the BHD and SHD, and those with no children < 5 and with fewer members (1-4) significantly (p < 0.05) used nets than the other groups.

**Table 4:** multinomial regression analysis of socio-demographic characteristics in association with LLINs use by all children < 5 and the entire household

DV →	Children < 5 years old‡ (n = 520)		Entire household‡ (n = 261)	
IV →	p-value	OR (95% C.I.)	p-value	OR (95% C.I.)
**Age groups (years)**				
20	⁑1.4x10-2	0.3 (0.1 - 0.8)	3.6x10-1	0.5 (0.1 - 2.0)
21-30	⁑9.0x10-3	0.5 (0.3 - 0.9)	⁑1.1x10-2	0.4 (0.2 - 0.8)
31-40	1.6x10-1	0.7 (0.5 - 1.1)	1.8x10-1	0.7 (0.4 - 1.2)
41-50	⁑7.6x10-3	0.5 (0.3 - 0.8)	2.6x10-1	0.7 (0.3 - 1.3)
51-60	Ref	1.0	Ref	1.0
**Gender**				
Female	7.5x10-2	0.7 (0.5 - 1.0)	4.0x10-1	†1.2 (0.8 - 1.8)
Male	Ref	1.0	Ref	1.0
**Marital status**				
Unmarried	⁑6.3x10-3	†1.5 (1.1 - 2.1)	6.3x10-2	†1.4 (1.0 - 2.1)
Married	Ref	1.0	Ref	1.0
**Education**				
NFE + Primary	⁑2.1x10-2	0.6 (0.4 - 0.9)	6.8x10-1	0.9 (0.6 - 1.5)
Secondary	1.0x10-1	0.7 (0.5 - 1.1)	1.9x10-1	0.7 (0.4 - 1.2)
Tertiary	Ref	1.0	Ref	1.0
**Occupation**				
Unemployed	9.8x10-2	0.6 (0.4 - 1.1)	7.8x10-2	0.5 (0.3 - 1.1)
Agricultural	⁑9.1x10-3	0.5 (0.3 - 0.9)	1.2x10-1	†1.5 (0.9 - 2.4)
Unskilled/Household/Domestic	3.6x10-1	0.8 (0.5 - 1.3)	6.5x10-1	†1.1 (0.7 - 2.0)
State/Parastatal	6.0x10-1	0.9 (0.5 - 1.5)	9.1x10-1	1.0 (0.6 - 1.9)
Professional	Ref	1.0	Ref	1.0
**Health District**				
Bamenda	⁑9.8x10-5	0.4 (0.3 - 0.6)	⁑8.1x10-17	0.1 (0.0 - 0.2)
Santa	⁑4.1x10-3	†2.1 (1.3 - 3.4)	⁑1.2x10-6	†15.1 (5.1 - 45.3)
Tiko	Ref	1.0	Ref	1.0
**House types**				
Caraboat	6.0x10-1	†1.2 (0.6 - 2.1)	6.8x10-1	0.9 (0.4 - 1.7)
Mixed (Block/Plank)	9.7x10-2	†1.6 (0.9 - 2.7)	8.0x10-1	†1.1 (0.5 - 2.8)
Mud Block	1.3x10-1	0.7 (0.5 - 1.1)	2.0x10-1	†1.4 (0.8 - 2.2)
Cement Block	Ref	1.0	Ref	1.0
**House size**				
1 - 3 bedrooms	6.8x10-1	0.9 (0.5 - 1.5)	⁑3.2x10-2	†2.0 (1.1 - 3.8)
4 - 7 bedrooms	Ref	1.0	Ref	1.0
**House composition**				
0 Children 0 - 5 SHLN	-	-	3.2x10-1	†1.3 (0.8 - 1.9)
1 - 2 Children 0 - 5 SHLN	-	-		
3 - 4 Children 0 - 5 SHLN	-	-	Ref	1.0
Family size 1 - 4	⁑2.4x10-22	†11.1 (6.8 - 18.1)	⁑1.0x10-12	0.1 (0.0 - 0.3)
Family size 5 - 7	5.5x10-1	†1.2 (0.7 - 1.8)	⁑2.2x10-3	0.3 (0.1 - 0.6)
Family size ≥ 8	Ref	1.0	Ref	1.0
**Own at least one LLINs**	⁑2.4x10-5	†23.1 (5.4 - 99.2)	⁑3.7x10-4	†40.5 (5.3 - 310.0)
**Install LLINs on all beds**	⁑1.4x10-4	†1.9 (1.4 - 2.6)	⁑2.6x10-4	†2.2 (1.4 - 3.3)
**Environmental factor**	⁑2.1x10-2	0.6 (0.3 - 0.9)	9.8x10-1	1.0 (0.6 - 1.7)

⁑: Statistically significant (p-value < 0.05), †: more likely possible, HH: Household, DV: Dependent variable, IV: Independent variable, SHLN: Slept Home Last Night, NFE: No Formal Education. ‡Reference category is: Yes.

Of the 1,251 households sampled, 484 (38.7%) regularly used bed nets on all nights of the week, while 350 (28.0%) had their household heads using bed nets last night (Table 2). The use of bed nets on all nights of the week and consequently the last night (Table 5) by the household head was associated to the age of the household head as well as to the health districts, where more household heads in the 21-30 and 31-40 age groups, and 20 and 41-50 age groups significantly (p < 0.05) used bed nets regularly and last night than the other age groups. Moreover, more household heads in the SHD and BHD significantly (p < 0.05) used bed nets on all nights of the week and last night, respectively (Table 5). The other uses, “out of the norms”, of LLINs are summarised in Table 6. Twenty-eight point seven percent (28.7%) (95% C.I; 26.3-31.3) of the household heads sampled, admitting that LLINs were put into other diverse uses. These uses ranged from being used as goal post nets by children; 2.8% (95% C.I; 2.0- 3.9), to yard fences; 22.7% (95% C.I; 20.5-25.1). Except for harvesting and drying of melon seeds (egussi), all the other “out of the norm” uses of LLINs were significantly (p < 0.05) associated to the health districts.

**Table 5:** multinomial regression analysis of socio-demographic characteristics in association with regular use of LLINs and house head use of LLINs last night

DV →	Regular use‡ (n = 484)		Slept under LLINs LN‡ (n = 484)	
IV →	p-value	OR (95% C.I.)	p-value	OR (95% C.I.)
**Age groups (years)**				
20	8.9x10-1	0.9 (0.4 - 2.5)	⁑4.7x10-2	0.4 (0.2 - 1.0)
21-30	⁑1.0x10-2	0.6 (0.4 - 0.9)	6.1x10-2	0.7 (0.4 - 1.0)
31-40	⁑6.3x10-3	0.5 (0.3 - 0.8)	⁑4.3x10-2	0.6 (0.4 - 1.0)
41-50	9.0x10-1	1.0 (0.6 - 1.6)	⁑2.8x10-2	0.6 (0.4 - 0.9)
51-60	Ref	1.0	Ref	1.0
**Gender**				
Female	7.6x10-1	1.0 (0.7 - 1.3)	9.6x10-1	1.0 (0.8 - 1.3)
Male	Ref	1.0	Ref	1.0
**Marital status**				
Unmarried	⁑6.0x10-3	†1.5 (1.1 - 2.0)	1.0x100	1.0 (0.8 - 1.3)
Married	Ref	1.0	Ref	1.0
**Education**				
NFE + Primary	8.4x10-1	1.0 (0.7 - 1.5)	⁑4.3x10-2	†1.4 (1.0 - 2.0)
Secondary	1.3x10-1	0.8 (0.5 - 1.1)	7.7x10-1	†1 (0.8 - 1.5)
Tertiary	Ref	1.0	Ref	1.0
**Occupation**				
Unemployed	7.0x10-1	0.9 (0.6 - 1.5)	8.9x10-1	1.0 (0.6 - 1.6)
Agricultural	1.8x10-1	0.7 (0.4 - 1.2)	7.3x10-1	†1.1 (0.7 - 1.7)
Unskilled/Household/Domestic	9.3x10-2	0.7 (0.4 - 1.1)	3.4x10-1	0.8 (0.5 - 1.3)
State/Parastatal	⁑2.3x10-2	0.6 (0.3 - 0.9)	5.7x10-1	0.9 (0.5 - 1.4)
Professional	Ref	1.0	Ref	1.0
**Health District**				
Bamenda	⁑1.5x10-9	†3.7 (2.4 - 5.7)	⁑3.5x10-2	0.6 (0.4 - 1.0)
Santa	⁑1.6x10-3	0.5 (0.3 - 0.8)	4.2x10-1	†1.2 (0.8 - 1.9)
Tiko	Ref	1.0	Ref	1.0
**House types**				
Caraboat	4.2x10-1	†1.3 (0.7 - 2.2)	⁑8.6x10-3	†2.5 (1.3 - 4.9)
Mixed (Block/Plank)	4.9x10-1	0.9 (0.5 - 1.3)	6.8x10-1	†1.1 (0.7 - 1.8)
Mud Block	⁑1.3x10-2	†1.6 (1.1 - 2.3)	7.6x10-1	†1.1 (0.8 - 1.5)
Cement Block	Ref	1.0	Ref	1.0
**House size**				
1 - 3 bedrooms	3.4x10-1	†1.3 (0.8 - 2.0)	8.2x10-1	0.9 (0.6 - 1.5)
4 - 7 bedrooms	Ref	1.0	Ref	1.0
0 Children 0 - 5 SHLN	⁑2.8x10-7	†2.3 (1.7 - 3.2)	2.6x10-1	0.8 (0.6 - 1.1)
1 - 2 Children 0 - 5 SHLN				
3 - 4 Children 0 - 5 SHLN	Ref	1.0	Ref	1.0
Family size 1 ï¿½ 4	2.1x10-1	0.7 (0.5 - 1.2)	5.6x10-2	†1.5 (1.0 - 2.4)
Family size 5 ï¿½ 7	6.0x10-1	0.9 (0.6 - 1.4)	1.5x10-1	†1.4 (0.9 - 2.1)
Family size ≥ 8	Ref	1.0	Ref	1.0
**Own at least one LLINs**	⁑7.1x10-3	†2.9 (1.3 - 6.2)	5.8x10-1	0.9 (0.5 - 1.5)
**Install LLINs on all beds**	⁑3.1x10-8	†2.4 (1.8 - 3.3)	1.2x10-1	†1.3 (0.9 - 1.7)
**Environmental factor**	8.7x10-1	1.0 (0.6 - 1.5)	6.9x10-1	0.9 (0.6 - 1.4)

⁑: Statistically significant (p value < 0.05), †: more likely possible, HH: Household, DV: Dependent variable, IV: Independent variable, SHLN: Slept Home Last Night, NFE: No Formal Education. ‡. Reference category is: Yes

**Table 6:** missuses of LLINs: by health district

S/N	Variable	Bamenda	Santa	Tiko	Total (%)	95% C.I.	χ^2^	p-value
	Aware of LLINs misuse	172	6	181	359 (28.7)	26.3-31.3	202.73	⁑9.5x10-45
	**Missuses**							
**1**	Football net	35	0	0	35 (2.8)	2.0 - 3.9	64.54	⁑9.7x10-15
**2**	Bathing/Bathroom shelter	23	29	5	57 (4.6)	3.5-5.9	19.04	⁑7.3x10-5
**3**	Fishing	32	11	15	58 (4.6)	3.6 ï¿½ 6.0	10.16	⁑6.2x10-3
**4**	Chicken shed/poultry	58	6	6	70(5.6)	4.5-7.0	71.40	⁑3.1x10-16
**5**	Harvesting/Drying egussi	46	40	26	112 (9.0)	7.5 - 10.7	5.76	5.6x10-2
**6**	Wall material	0	125	3	127 (10.2)	8.7 - 12.0	299.50	⁑9.2x10-66
**7**	Mesh on windows	83	27	74	184 (14.7)	12.9 - 16.8	26.37	⁑1.9x10-6
**8**	Yard/Garden fences	153	79	52	284 (22.7)	20.5 - 25.1	59.60	⁑1.1x10-13

⁑: Statistically significant (p-value < 0.05)

**Care and maintenance of LLINs:** out of the 1,251 household heads sampled, 1,089 (87.1%) said LLINs can be washed, while 634 (50.7%) affirmed the recommended LLINs washing frequency of 2-4 times a year. The question of washing bed nets or not (Table 7), was associated to the age of the household head, occupation, and health district, where households with heads in the age range 21-50 years, unemployed and unskilled, and those in the BHD significantly (p < 0.05) washed them compared to those in the other groups. On the recommended LLINs washing frequency, heads in the BHD (p = 6.2x10-5), OR; 2.0, 95% C.I; 1.4-2.8) were significantly more likely, while those in the SHD (p = 6.3x10-1), OR; 1.1, 95% C.I; 0.8-1.6) were insignificantly more likely to respect the recommended LLINs washing frequency compared to those in the THD (Table 6).

**Table 7:** regression analysis of socio-demographic characteristics in association with LLINs maintenance

DV →	Can wash LLINs‡ (n = 1089)		Recommended wash frequency‡ (n = 634)	
IV →	p-value	OR (95% C.I.)	p-value	OR (95% C.I.)
**Age groups**				
20	2.1x10-1	†1.9 (0.7 - 5.5)	8.0x10-1	0.9 (0.4 - 2.0)
21 – 30	3.5x10-1	†1.3 (0.7 - 2.3)	1.4x10-1	†1.3 (0.9 - 1.9)
31 – 40	3.8x10-1	†1.3 (0.7 - 2.2)	1.6x10-1	†1.3 (0.9 - 1.9)
41 – 50	9.7x10-1	1.0 (0.5 - 1.9)	1.5x10-1	†1.3 (0.9 - 2.0)
51 – 60	Ref	1.0	Ref	1.0
**Gender**				
Female	⁑9.2x10-3	0.6 (0.4 - 0.9)	9.8x10-1	1.0 (0.8 - 1.3)
Male	Ref	1.0	Ref	1.0
**Marital status**				
Unmarried	2.4x10-1	†1.2 (0.9 - 1.8)	5.4x10-1	0.9 (0.7 - 1.2)
Married	Ref	1.0	Ref	1.0
**Education**				
NFE + Primary	8.9x10-1	1.0 (0.6 - 1.5)	6.9x10-1	†1.1 (0.8 - 1.4)
Secondary	2.7x10-1	0.8 (0.5 - 1.2)	4.9x10-1	0.9 (0.7 - 1.2)
Tertiary	Ref	1.0	Ref	1.0
**Occupation**				
Unemployed	3.5x10-1	†1.4 (0.7 - 2.6)	2.7x10-1	†1.3 (0.8 - 1.9)
Agricultural	7.4x10-1	†1.1 (0.6 - 1.9)	9.3x10-1	1.0 (0.7 - 1.4)
Unskilled/Household/Domestic	8.2x10-1	†1.1 (0.6 - 1.9)	5.7x10-1	†1.1 (0.8 - 1.7)
State/Parastatal	4.4x10-1	†1.3 (0.7 - 2.4)	5.8x10-1	0.9 (0.6 - 1.4)
Professional	Ref	1.0	Ref	1.0
**Health District**				
Bamenda	1.1x10-1	†1.5 (0.9 - 2.5)	⁑6.2x10-5	†2.0 (1.4 - 2.8)
Santa	9.8x10-1	1.0 (0.6 - 1.7)	6.3x10-1	†1.1 (0.8 - 1.6)
Tiko	Ref	1.0	Ref	1.0


⁑: Statistically significant (p-value < 0.05), †: more likely possible, HH: Household, DV: Dependent variable, IV: Independent variable, SHLN: Slept Home Last Night, NFE: No Formal Education. ‡. Reference category is: Yes

## Discussion

This study examined the indicators of LLINs ownership/utilisation and maintenance in the Bamenda, Santa and Tiko Health Districts. Overall, 92.5%, 20.5% of the households interviewed owned at least one LLIN per household and utilisation by the entire household last night, respectively.

Indicators of household LLINs ownership: currently, the targets in national strategic plans for all three LLINs coverage indicators are usually set for all people at risk of malaria [1,2,29], to ≥ 80%. Household ownership of at least one LLIN per household in this study is higher than rates reported elsewhere in Cameroon [6,7,17-19] and out of Cameroon [30-34]. It was however, lower than the proportions reported in Uganda and Myanmar [29,35], and in line with the 93.5% reported in Madagascar [31]. The high proportion of owning at least a LLIN per household in these health districts could be attributed to the free LLINs mass distribution campaign (MDC) [6,12]. The universal household coverage of 66.8% (overall LLIN: person ratio of 0.50), although within the WHO range of 39-75 % [36], was lower than rates reported elsewhere in Cameroon and Myanmar [15,29]. It was however, higher compared to rates in Madagascar, DRC, and Uganda [31,37,38] as well as a host of eight African countries [32]. Access to LLINs in the household of 65.5% in this study was lower compared to results reported elsewhere [31,35,38], higher than the 21% reported in Batwa [39], within the 57.3-78.8% in eight African countries [32] and 32.3-81.3% reported in a multi-country study [40]. The low household universal coverage and (versus) accessibility in this study could be attributed to the significant differences amongst the health districts: 86.4% vs 81.0% for BHD, 55.6% vs 47.0% for SHD and 56.2% vs 65.8% for THD; and differences in family size vs gender of household head. The significant association of universal coverage and accessibility to health districts is in line with a study in Uganda [41].

**Household utilisation of LLINs:** household universal LLINs utilisation of 20.5% (16.0% of the de facto population) was very low compared to previous studies reported elsewhere in Cameroon [18,19] and out of Cameroon [31,35,37,42,43]. This was however high compared to the 6.9- 15.3% reported in Myanmar [30]. The very low household LLINs utilisation could be attributed to the significant differences recorded amongst the health districts: 43.1% in the BHD, 1.0% in the SHD, and 14.1% in the THD; as well as household composition and the installation of LLINs on all beds in the household. It could also be due to inadequate education on LLINs utilisation, socio-political tensions, and differences in the different study designs. Bed-net utilisation by all children 0-5 years and expectant mothers in the household of 14.6% and 63.4%, respectively, is low compared to 63% vs 60% reported in the BHD [18], 52% vs 58% in the national territory [7] and elsewhere in the world [29,31,34,42,44]. The low LLIN utilisation by all children 0-5 years old could be attributed to significant differences in the health districts, age of household heads, educational status of the household head as well as the presence or absence of bushes or water pools around dwellings (p < 0.05). Use of LLINs by the household head last night of 28.0% was low compared to 58.3% reported in Rural and semi-Urban communities in the South West Region of Cameroon [6] and 47.2% in China [27]. Meanwhile, the regular use of LLINs of 38.7% was low compared to 48.0% reported in China [27]. The low use of LLINs last night by the household head and regular use of LLINs could significantly be attributed to differences in the health districts and age of the household heads (p < 0.05). LLIN misuse of 2.3-22.7% was also similar to the 18.2% reported in Mezam Division [17] and 21% in Kenya [45]. The use of LLINs for other purposes, other than the prevention of mosquito bites could be attributed to: inadequate education on utilisation, lack of good playgrounds as 2.8% (95% C.I, 2.0-3.9) of the households admitted that children used LLINs as football goal post nets.

**Care and maintenance of LLINs:** out of the 1,251 household heads sampled, 1,089 (87.1%) said LLINs can be washed, while 634 (50.7%) affirmed the recommended LLIN washing frequency of 2-4 times a year. The recommended LLIN wash frequency reported in this study was similar to the 52.0% reported in Kenya [45] and lower than the 57.7-77% in Côte d'Ivoire and Western Kenya [20,28]. The optimal LLINs washing frequency could be attributed to the gender of the household head as well as the health district.

**Recommendations:** the populations of the three health districts as well as the entire national territory, should be properly educated by community health workers and stakeholders on the regular utilisation of LLINs and by all household occupants. The MOH should sustain another free MDC since those distributed in 2015 - 2016 will be worn out and ineffective in preventing malaria by 2019.

### Strengths and limitations of the study

**Strengths:** the data used in this study was collected by trained surveyors, who had mastery of the HAs as they are responsible for the coding of houses during the Expanded Programme on Immunisation (EPI) and MDC campaigns. The health district offices were consulted for the mapping of the HAs, quarters and census lists of households used in the last MDC and EPI campaigns. In Cameroon, the MOH carries out seasonal EPI campaigns. The quality of data collected was assured through the multistage sampling strategy to minimize bias and pretesting of questionnaires.

**Limitations:** this was a cross-sectional study, representing a snapshot of the population within the study period and does not show cause and effect since the predictor and outcome variables were measured at the same time. Data was collected through self-reporting and thus there is a possibility of bias where the respondent provides socially acceptable answers. Recall bias can also affect some of the responses and subsequently the results of the study. In this study, however, respondents were required to only recall whether they and occupants of their households slept under a LLIN the previous night, as well as the source and number of LLINs in the household. Ownership and number of LLINs in the household was not evaluated. Ownership, utilisation, and maintenance of LLINs in the three health districts in 2017 and 2018 could not be attributed solely to the 2015 - 2016 MDC, as other sources of LLINs: ANC for pregnant women and gift from a relation, and our study design could not capture the contribution of each intervention.

## Conclusion

Our findings highlighted low rates of household universal coverage, accessibility, and utilisation indicators as well as maintenance amidst high ownership of at least one LLIN per household and free MDC. However, efforts had reached vulnerable populations in all health districts. Quelling the on-going socio-political crisis and scaling up efforts can lead to increased coverage which may systematically contribute to household universal utilisation and thus reduce malaria morbidity and mortality. Our finding that health districts are strongly associated with LLINs ownership, utilisation and maintenance suggests that MDCs should be complemented by education and behaviour change communication, emphasizing that malaria is transmitted by mosquito bites and it can be prevented by sleeping under LLINs.

### What is known about this topic

- *Long-lasting insecticide nets are now in use as a means to control and reduce the incidence of malaria;*
- *Mass distribution campaigns are organised periodically to continually curb malaria*.

### What this study adds

- *The ownership of LLINs does not necessarily mean its utilisation;*
- *The knowledge on the care and maintenance of LLINs*.
